# Enhancing reaction rate in a Pickering emulsion system with natural magnetotactic bacteria as nanoscale magnetic stirring bars†
†Electronic supplementary information (ESI) available. See DOI: 10.1039/c7sc05164f


**DOI:** 10.1039/c7sc05164f

**Published:** 2018-01-31

**Authors:** Xin Zhou, Changyou Chen, Changyan Cao, Tao Song, Hengquan Yang, Weiguo Song

**Affiliations:** a Beijing National Laboratory for Molecular Sciences , Laboratory of Molecular Nanostructures and Nanotechnology , CAS Research/Education Center for Excellence in Molecular Sciences , Institute of Chemistry , Chinese Academy of Sciences , China . Email: cycao@iccas.ac.cn ; Email: wsong@iccas.ac.cn; b University of Chinese Academy of Sciences , Beijing100049 , China; c Institute of Electrical Engineering , Chinese Academy of Sciences , Beijing 100190 , China; d School of Chemistry and Chemical Engineering , Shanxi University , Taiyuan 030006 , China

## Abstract

Pickering emulsion is emerging as an advanced platform for catalysis because of the large oil/water interface area for reaction and its superior efficiency. How to enhance the mass transportation within the micro-droplets is the biggest obstacle in further improving the efficiency of the Pickering emulsion system. In this study, we propose and solve this problem for the first time using natural magnetotactic bacteria as nanoscale magnetic stirring bars, which can be encapsulated into each micro-droplet and used to stir the solution to accelerate the mass transportation under an external magnet, and thus significantly enhance the reaction rate of Pickering emulsion. Taking the epoxidation of cyclooctene in the Pickering emulsion system as a demonstration, the reaction rate was enhanced three times with nanoscale magnetic stirring bars compared to that of traditional Pickering emulsion, and was even thirty times higher than that of conventional stirrer-driven biphasic systems. We envision that this strategy will bring biphasic reactions with fundamental innovations toward more green, efficient and sustainable chemistry.

## Abstract 2

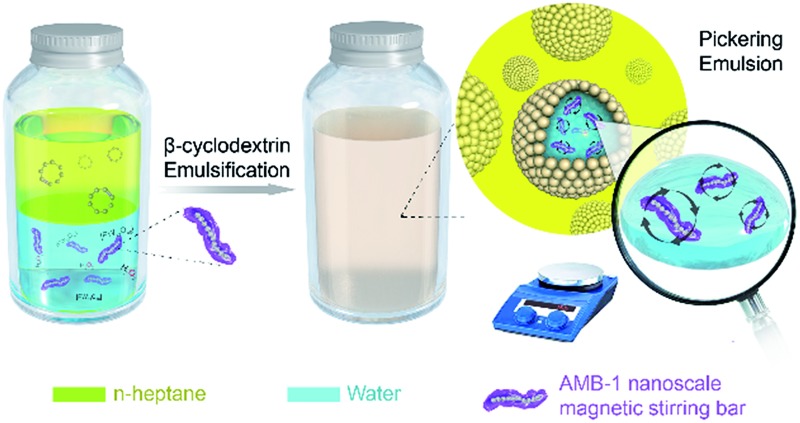
The reaction rate of Pickering emulsions was greatly enhanced with natural magnetotactic bacteria as nanoscale magnetic stirring bars.

## Introduction

During the past two decades, sustainable chemistry has been a very important topic for its advantages, such as being green and highly efficient.^1^ A green and highly efficient chemical process should have the following features: (1) a minimum amount of byproducts and wastes, (2) safe and benign solvents and reactants, and (3) a high efficiency of mass transportation and catalytic processes.^2^ The Pickering emulsion system seems to be one of these processes.^3^ Nardello-Rataj^2^,^4^ and Yang^5^*et al.* designed static Pickering emulsion systems for catalytic reaction and observed excellent efficiency, proving the great potential of the Pickering emulsion system in catalysis. In a Pickering emulsion, each droplet in the emulsion can be considered as a micro-reactor, which provides a large interface for reaction and enhances the mixing of catalysts and reactants. However, the reaction solution is static within each droplet, *i.e.* the mass transportation inside the droplet is driven by only the concentration gradient. Thus, the catalytic reaction rate is still limited by mass transportation within the droplets. How to enhance the mass transportation efficiency within the droplets is the biggest obstacle in further improving the efficiency of the Pickering emulsion system. In this study, we solved this problem using nanoscale magnetic stirring bars.

Magnetic stirring and mechanical stirring are often used to accelerate the reaction rate in conventional bottle reaction systems. However, these conventional stirring methods are impractical for micro-droplets^6^ or Pickering emulsions.^2^,^3^,5f Nanoscale magnetic stirring bars have also been reported.^7^ Chen’s group^8^ proposed 1D assembly nanoparticles and nanometer-sized magnetic stirring bars, which are small enough to be used for stirring inside extremely small droplets under an external magnetic field. In our previous work,^9^ we demonstrated that nanoscale magnetic stirring bars can be used in an array of microdroplet systems to boost the catalytic activity. We envision that the small size as well as magnetic response characteristic of nanoscale magnetic stirring bars provides the possibility to stir the droplets in Pickering emulsion.

To date, only very few kinds of nanoscale magnetic stirring bar have been reported and the fabrication procedures were complicated and time-consuming.8a,^9^ Therefore, it is also highly desirable to develop a facile and efficient way to prepare new kinds of nanoscale magnetic stirring bar. Magnetotactic bacteria (MTBs)^10^ are a group of polyphyletic microorganisms with a broad range of morphological types, which are able to biomineralize intracellular nano-sized magnetosomes of Fe_3_O_4_ (magnetite) and Fe_3_S_4_ (greigite) and align them in a linear manner.^11^ By organizing magnetosomes into a chain-like structure, MTBs can navigate along geomagnetic field lines. They will also migrate following an external magnetic field.^12^ Because of their uniform genetically controlled nano-morphology, narrow size distributions and bio-membrane coating, magnetosomes are better than artificial iron-oxide nanochains.^13^ So far, researchers have separated and cultured dozens of kinds of MTB, such as MSR-1, AMB-1, MGT-1, *etc.*,11a,d offering a large variety of MTBs for fabrication of nanoscale magnetic stirring bars.

Herein, we demonstrated for the first time that the reaction rate of Pickering emulsion was indeed significantly enhanced with natural MTBs as nanoscale magnetic stirring bars, which can be encapsulated into each micro-droplet and used to stir the solution to accelerate the mass transportation under an external magnet. Taking the epoxidation of cyclooctene in the Pickering emulsion system as a test model, the reaction rate with nanoscale magnetic stirring bars was three times higher than that of traditional static Pickering emulsion, and in striking comparison, thirty times higher than that of conventional stirrer-driven biphasic systems under the same conditions.

## Experimental

### Materials

AMB-1 was provided by Song’s group from IEECAS (Institute of Electrical Engineering Chinese Academy of Science), cyclooctene (>95%) was provided by TCI (Shanghai) Development Co., Ltd., HCl (36–38%), *n*-heptane (A.R.) and H_2_O_2_ (30%) were provided by Beijing Chemical Reagent Company, β-cyclodextrin was provided by SCRC (Sinopharm Chemical Reagent Co.,Ltd), Na_3_PW_12_O_40_ was provided by Alfa-Aesar, *o*-tolidine (98%) and glutaraldehyde (50 wt%) were provided by J&K Scientific Co.,Ltd, PBS solution was provided by Boster (AR0030), and SYTO 9 (L13152) was provided by Thermo Fisher Scientific.

### Preparation of AMB-1 nanoscale magnetic stirring bars

AMB-1 was collected from culture medium and washed with PBS–buffer solution three times. Then the bacteria were dispersed in glutaral–PBS solution (2.5 wt%) with a concentration of 1.5 mg ml^–1^. The mixture was kept at 4 °C for 24 h, and washed with deionized water 3 times again. Finally, the concentrated AMB-1 was dispersed in deionized water with a concentration of 1.5 mg ml^–1^ and kept at 4 °C (denoted DMTB).

### Fluorescence labeling of AMB-1

SYTO 9 was dissolved in 1 ml of AMB-1 solution with a certain ratio, then the mixture was kept static for 15 min and kept out of the light. After AMB-1 was fully integrated with the dye, the solution was mixed with Na_3_PW_12_O_40_ and an oil phase as above mentioned to fabricate the PE system.

### The epoxidation reaction of cyclooctene with normal stirring

0.1767 g of Na_3_PW_12_O_40_ was dissolved in 2 ml of deionized water and 0.4 ml of H_2_O_2_ (30%) was added in the solution. Then 0.26 ml of cyclooctene was mixed with 2 ml of *n*-heptane, after which the water phase and oil phase were added in a 50 ml reactor with a normal stirring bar. The reactor was put in a 45 °C water bath with a certain speed of rotating magnetic field. The product was analyzed by a gas chromatograph (Shimadzu GC-2010) equipped with a flame ionization detector (FID) and a Rtx-5 capillary column (0.25 mm in diameter, and 30 m in length).

### The epoxidation reaction of cyclooctene in the Pickering emulsion system

0.1767 g of Na_3_PW_12_O_40_ was dissolved in 2 ml of deionized water and 0.4 ml of H_2_O_2_ (30%) was added in the solution. Then 0.26 ml of cyclooctene was mixed with 2 ml of *n*-heptane, after which the water phase and oil phase, and 0.5 g of β-cyclodextrin, were added in a 50 ml reactor, and were emulsified by an Ultra-Turrax instrument (8000 rpm, IKA, T25) for 60 s. The emulsion was kept static in a 45 °C water bath under the same speed of rotating magnetic field as a normal stirring system and the product was analyzed by GC.

### The epoxidation reaction of cyclooctene in the Pickering emulsion system with AMB-1

0.1767 g of Na_3_PW_12_O_40_ was dissolved in 2 ml of AMB-1 solution and 0.4 ml of H_2_O_2_ (30%) was added in the solution. Then 0.26 ml of cyclooctene was mixed with 2 ml of *n*-heptane, after which the water phase and oil phase. and 0.5 g of β-cyclodextrin, were added in a 50 ml reactor, and were emulsified by an Ultra-Turrax instrument (8000 rpm, IKA, T25) for 60 s. The emulsion was kept static in a 45 °C water bath under the same speed of rotating magnetic field as the above two systems and the product was analyzed by GC, too.

### Test of the variation in concentration of H_2_O_2_ solution with/without AMB-1 nanoscale magnetic stirring bars

1 ml of 30% H_2_O_2_ solution was added to 2 ml of AMB-1 solution, and taken as a control group, and 1 ml of 30% H_2_O_2_ solution was added to 2 ml of deionized water. Both of the samples were placed in a 45 °C water bath. The variation in concentration of H_2_O_2_ was detected by UV-vis spectroscopy (Shimadzu, UV-2550) using *o*-tolidine as the peroxide indicator.^14^ For each test, 0.5 ml of reaction suspension was diluted to 2 ml after being taken from the system and the supernatant was collected after centrifugation. A 0.5 mL volume of 1% *o*-tolidine in 0.1 M HCl was added to the supernatant. This mixture was allowed to react for 2 min. Subsequently, the dispersion was acidified with 1 M HCl (2 ml), which caused the color of the dispersion to turn yellow. The yellow-coloured species is the protonated form of the 2-electron oxidation product of *o*-tolidine that was formed. The absorption spectrum of the 2-electron oxidized *o*-tolidine has a characteristic maximum at 438 nm.

### Characterization

The optical and fluorescence images were characterized by laser scanning confocal microscopy (LSCM, OLYMPUS FV1200-IX83). The morphology and microstructures of the samples were characterized by transmission electron microscopy (TEM, JEOL, JEM-2100F) and high-resolution transmission electron microscopy (TEM, JEOL, JEM-2100F). Magnetic properties of AMB-1 were tested by MPMS XL (Quantum Design) with a magnetic field from 0.6 T to –0.6 T at 300 K with a step of 0.005 T. The water contact angle (WCA) was performed on a contact angle analyzer (SPCA-2, HARKE). Magnetic rotation speed and reaction temperature were controlled by a magnetic stirrer (IKA, RCT basic).

## Results and discussion

### Design and fabrication of the cyclooctene Pickering emulsion system

**Scheme 1:**
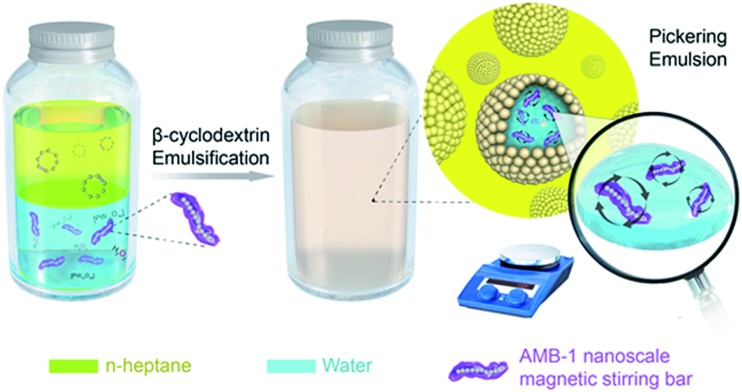
Formation processes of the cyclooctene Pickering emulsion system containing nanoscale magnetic stirring bars.

Scheme 1 shows the formation processes of the cyclooctene Pickering emulsion system containing nanoscale magnetic stirring bars. Firstly, a water phase containing an Na_3_PW_12_O_40_ catalyst and H_2_O_2_ oxidant as well as nanoscale magnetic stirring bars, and an oil phase composed of cyclooctene in *n*-heptane solution are prepared separately. After adding β-cyclodextrin as an emulsifier, the mixture is emulsified under high rotation to form the stable water-in-oil Pickering emulsion. Because the nanoscale magnetic stirring bars are small enough and hydrophilic, they can be well dispersed in the water phase. With a good magnetic response to the external rotating magnetic field, they can stir the water solution inside each water phase droplet. Thus, mass transfer efficiency in each water micro-droplet is significantly enhanced, resulting in an enhanced reaction rate of epoxidation of cyclooctene in Pickering emulsion.

### Characterization of the nanoscale magnetic stirring bars

**Fig. 1:**
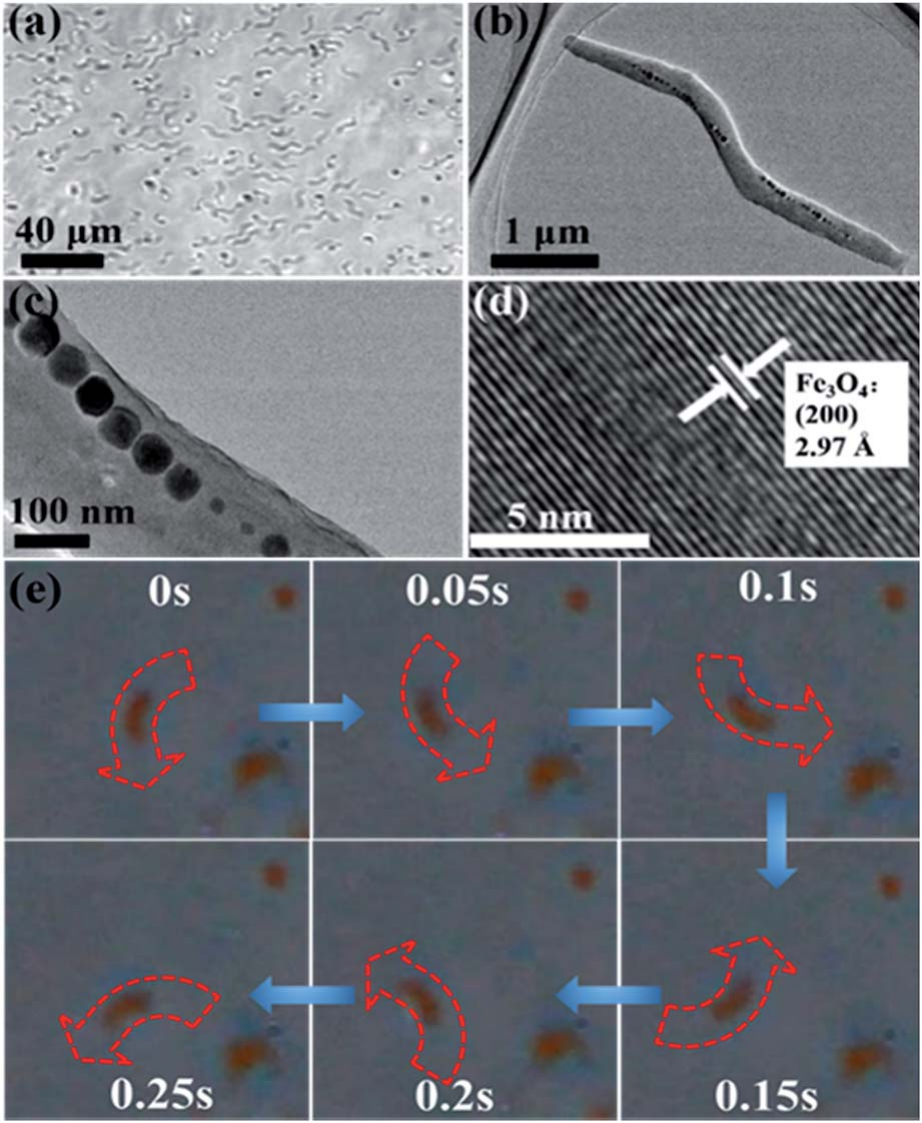
(a) Laser scanning confocal microscope image, (b and c) TEM images of a single chain/whole cell, (d) HRTEM image of a magnetosome of AMB-1 magnetotactic bacteria after immobilization with glutaral–phosphate buffered saline solution, and (e) snapshot micrographs of the rotational angles of one typical nanoscale magnetic stirring bar under an external magnet.

AMB-1 is one kind of magnetotactic bacteria that contains straight-line chains inherently assembled by Fe_3_O_4_ nanoparticles. An individual AMB-1 bacterium is like a strip of 2–3 μm in length. Inside the body of AMB-1 is a chain composed of dozens of magnetosomes (Fe_3_O_4_, Fig. 1d), which have a size of ∼50 nm in diameter.^15^ This feature and structure of AMB-1 is very suitable for preparing nanoscale magnetic stirring bars. AMB-1 magnetotactic bacteria were first immobilized with glutaral–phosphate buffered saline solution to preserve the morphology and rigid structure (Fig. 1a–c). One important reason for immobilization of the whole magnetotactic bacteria is that an individual magnetosome chain without a cell membrane or a cell wall is apt to collapse and aggregate, leading to a low magnetic response.11c,d,^13^,^15^,^16^ Immobilizing the bacteria also passivates the surface of the bacteria, so that the surface of the bacteria will not be involved in the catalytic reaction.

We next investigated the magnetic properties of the obtained nanoscale stirring bars. The hysteresis loops were measured at 300 K. Fig. S1† shows hysteretic properties for the bacteria sample with a coercivity of 19.0 mT (*B*_c_), and a *B*_cr_ (coercivity remanence) value of 21.6 mT, while the value of *M*_rs_ (saturation remanence magnetization)/*M*_s_ (saturation magnetization) is 0.44. Due to the above magnetic properties of immobilized AMB-1 stirring bars, they can stir the aqueous solution well under an external magnetic field. Fig. 1e shows the snapshots of the stirring behaviors of one typical nanoscale magnetic stirring bar under an external magnet. The quick rotation of the AMB-1 based stirring bars could be readily observed and recorded under a microscope. It can be seen that the angle of the bacteria changes at different times. The detailed stirring behavior is shown in Video S1.† The facile preparation procedure and excellent stirring properties make magnetotactic bacteria an ideal source for constructing nanoscale magnetic stirring bars directly.

### Characterization of the cyclooctene Pickering emulsion system with nanoscale magnetic stirring bars

**Fig. 2:**
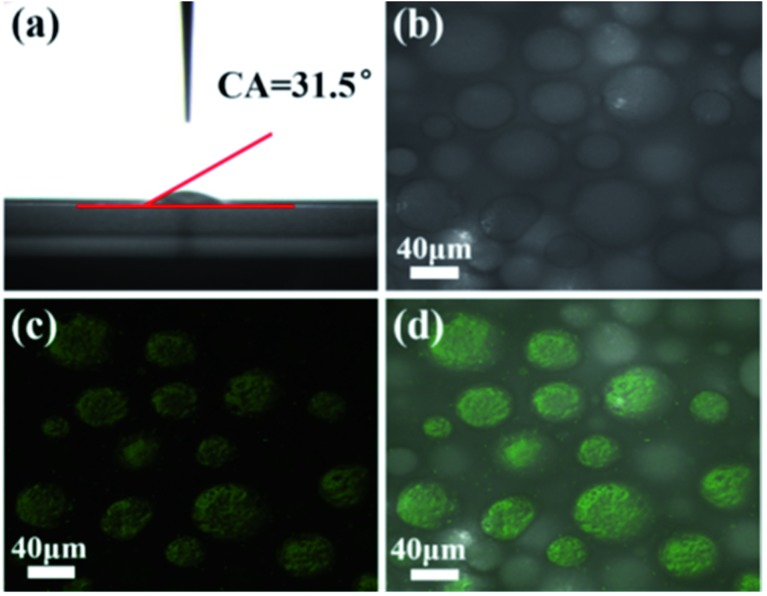
(a) Water contact angle, and (b–d) laser scanning confocal microscope images of the Pickering emulsion containing nanoscale magnetic stirring bars. The regions in green are water droplets containing fluorescence labelled AMB-1.

Due to the intrinsic properties of the cell membrane and wall of AMB-1 magnetotactic bacteria, the immobilized nanoscale magnetic stirring bars showed hydrophilic properties with a water contact angle of about 31.5° (Fig. 2a). Thus, these nanoscale magnetic stirring bars can be dispersed well in the water phase. After adding β-cyclodextrin as an emulsifier, a stable water-in-oil Pickering emulsion with diameters of the micro-droplets of about 30–80 μm was formed (Fig. 2b). In order to confirm the location of nanoscale magnetic stirring bars in the emulsion system, a fluorescence labelling method was used. As shown in Fig. 2c, the labelled bacteria showed green light under an excitation wavelength of 488 nm. The overlap of the positions of the micro-droplets and the fluorescence from labelled materials confirmed that nearly all the nanoscale magnetic stirring bars were inside the water phase (Fig. 2d). The sizes of the magnetic stirring bars were much smaller than that of the micro-droplets, and they can stir the water solution under an external magnetic field, which is very useful for enhancing the mass transfer efficiency of the water-phase in the Pickering emulsion.

### Catalytic performance of the nanoscale magnetic stirring bars in the Pickering emulsion system

**Fig. 3:**
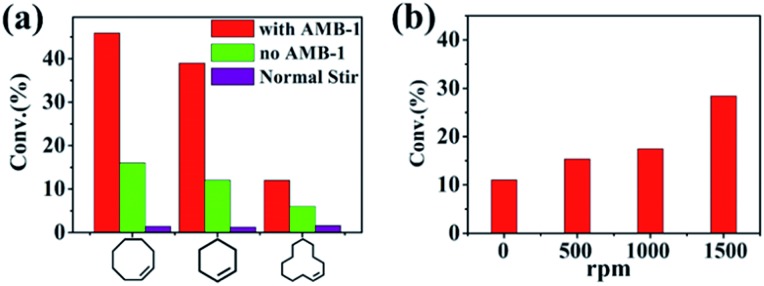
(a) Comparison of conversions for different substrates under three reaction systems, and (b) conversions of epoxidation of cycloocetene with different magnetic rotation speeds in the Pickering emulsion system containing nanoscale magnetic stirring bars.

With the Pickering emulsion system containing nanoscale magnetic stirring bars, we examined the epoxidation of cyclooctene in a static Pickering emulsion system under an external magnetic field. For comparison, a normal two-phase reaction system with traditional magnetic stirring bars and a static Pickering emulsion system without nanoscale magnetic stirring bars were also tested. Fig. 3a shows the conversions of cyclooctene at 45 °C for 2 hours under three different reaction systems. The system with normal magnetic stirring bars showed the lowest conversion of cyclooctene of about 1.4%. Such low conversion was due to it having the lowest interface area of the boundary between the two phases. The conversion of cyclooctene was increased to about 13.4% with the static Pickering emulsion system, confirming the advantage of the Pickering system. In striking comparison, the cyclooctene conversion was boosted to about 45.9% with 99% selectivity for the Pickering emulsion system with nanoscale magnetic stirring bars when an external magnetic field was applied (the stirring speed was set as 1500 rpm on the magnetic plate). The reaction rate of the Pickering emulsion with nanoscale magnetic stirring bars is three times higher than that of the traditional static Pickering emulsion and even thirty times higher than that of the conventional stirrer-driven biphasic system under otherwise identical conditions. Besides cyclooctene, the reaction rates for cyclohexene and cyclododecene can also be greatly enhanced in the Pickering emulsion system with nanoscale magnetic stirring bars, demonstrating the general usefulness of this strategy.

The enhanced catalytic efficiency of the Pickering emulsion with nanoscale magnetic stirring bars can be directly ascribed to the enhancement of mass transfer efficiency in the water phase. In order to confirm this, we first need to confirm that nanoscale magnetic stirring bars (glutaral immobilized AMB-1 bacteria) are not involved in the reaction. TEM images showed that nanoscale magnetic stirring bars maintained their original rigid chain structure even after being immersed in H_2_O_2_ for 5 h (Fig. S2†). Furthermore, we investigated the decomposition rates of H_2_O_2_ with/without nanoscale magnetic stirring bars under the reaction temperature (45 °C). The results showed that the decomposition rates of H_2_O_2_ were nearly the same (Fig. S3†), indicating that the nanoscale magnetic stirring bars had no effect on H_2_O_2_ decomposition and were not involved in the reaction.

The enhancement of mass transfer efficiency in the Pickering emulsion with nanoscale magnetic stirring bars was then further confirmed through changing the stirring speeds from 0 to 1500 rpm. The results showed an obvious difference of conversions for cycloocetene in 1 h (Fig. 3b). The conversion increased with the increase of the rotation speed. Under higher stirring speeds, the rotation in the water micro-droplets caused by the nanoscale stirring bars was more obvious, leading to better mass transfer efficiency and a higher reaction rate. It needs mentioning particularly that when the external magnetic field was turned off (the stirring speed was 0 rpm), the conversion of cycloocetene was about 11%, which was nearly the same as that of the static Pickering emulsion without adding the nanoscale magnetic stirring bars (Fig. S4†). This result also suggested that the nanoscale magnetic stirring bars had no catalytic activity for conversion of cyclescene. Thus, we can conclude that the enhanced reaction rate of the Pickering emulsion with nanoscale magnetic stirring bars should be attributed to the enhancement of mass transfer efficiency in the water phase under an external magnetic field.

**Fig. 4:**
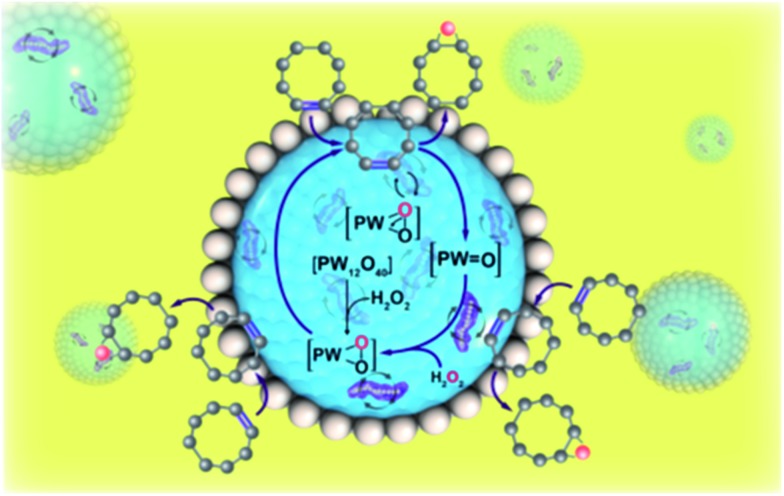
Scheme illustration of the reaction in the Pickering emulsion system with nanoscale magnetic stirring bars.

Fig. 4 illustrates the proposed reaction scheme in the Pickering emulsion system containing nanoscale magnetic stirring bars. In a typical Pickering emulsion, the reaction occurs at the oil/water interface. In the water phase, the polyoxometalate reacts with hydrogen peroxide to form polyoxometalate active species, which are then reacted with cyclooctene at the oil/water interface.^8^ Due to the huge oil/water interface area of the emulsion system, the reaction rate is enhanced compared to the normal two-phase reaction system with traditional magnetic stirring bars. When the nanoscale magnetic stirring bars are added to the water-phase and an external magnetic field is applied, the mass transfer efficiency within the water micro-droplet is further greatly enhanced. Therefore, the formation rate of polyoxometalate active species and the following diffusion rate to the oil/water interface are significantly enhanced, boosting the reaction rate of Pickering emulsion.

As mentioned earlier, the surface of the bacteria was passivated in order not to interfere with the epoxidation reaction in this study, as the Na_3_PW_12_O_40_ catalyst was dissolved in the water phase. In future studies, we envision that the surface of the bacteria could be loaded with catalyst nanoparticles for other heterogeneous catalytic reaction systems, making the bacteria based stirring bars act as catalyst carriers. Such exploration is underway in our lab.

## Conclusions

In summary, we demonstrated that the reaction rate of a Pickering emulsion system can be significantly enhanced with nanoscale magnetic stirring bars, which were produced directly from magnetotactic bacteria. The nanoscale magnetic stirring bars were encapsulated into each micro-droplet and used to stir the solution to accelerate the mass transportation under an external magnetic field. The reaction rate for epoxidation of cyclooctene using the nanoscale magnetic stirring bars was three times higher than that of traditional static Pickering emulsion and even thirty times higher than that of a conventional stirrer-driven biphasic system under the same conditions. In view of the usefulness of the nanoscale magnetic stirring bars in Pickering emulsions, we envision that this strategy will bring biphasic reactions with fundamental innovations toward more green, efficient and sustainable chemistry.

## Conflicts of interest

There are no conflicts to declare.

## Supplementary Material

Supplementary information

Supplementary movie
