# Supplementary material for: Enhancing reaction rate in a Pickering emulsion system with natural magnetotactic bacteria as nanoscale magnetic stirring bars
Source: Chem Sci. 2018 Jan 31;9(9):2575–80. doi: 10.1039/c7sc05164f (PMC5897955; doi:10.1039/c7sc05164f)
Supplement: Supplementary file 1 [file SC-009-C7SC05164F-s001.pdf]

Electronic Supplementary Material (ESI) for Chemical Science.
This journal is © The Royal Society of Chemistry 2018

Electroni Supplementary Information(ESI)

Enhancing reaction rate in Pickering emulsion system with natural magnetotactic bacteria as nanoscale magnetic stirring bars

Xin Zhou,[a, b] Changyou Chen,[c] Changyan Cao, *[a, b] Tao Song,[c] Hengquan Yang[d] and Weiguo Song*[a, b]

[a] Beijing National Laboratory for Molecular Sciences, Laboratory of Molecular Nanostructures and Nanotechnology CAS Research/Education Center for Excellence in Molecular Sciences, Institute of Chemistry, Chinese Academy of Sciences, Beijing 100190 (China). *E-mail: cycao@iccas.ac.cn, wsong@iccas.ac.cn

[b] University of Chinese Academy of Sciences, Beijing100049 (China)

[c] Institute of electrical engineering, Chinese Academy of Sciences, Beijing 100190 (China). *E-mail: songtao@mail.iee.ac.cn

[d] School of Chemistry and Chemical Engineering, Shanxi University, Taiyuan 030006 (China). *E-mail: hqyang@sxu.edu.cn

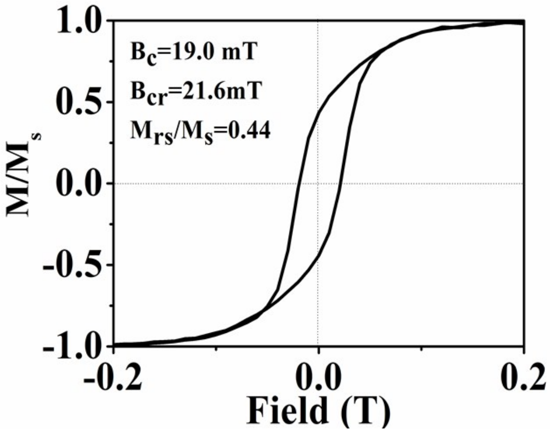

Fig. S1 Hysteretic properties for the bacteria sample.

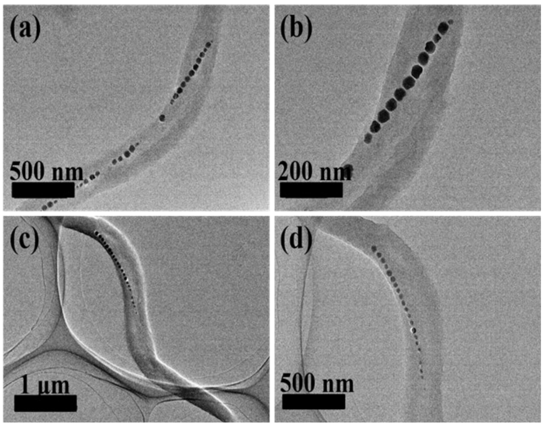

Fig. S2 TEM images of AMB-1 cell after immersed in 5% H_2O_2 solution for (a, b) 3 hours and (c, d) 5 hours.

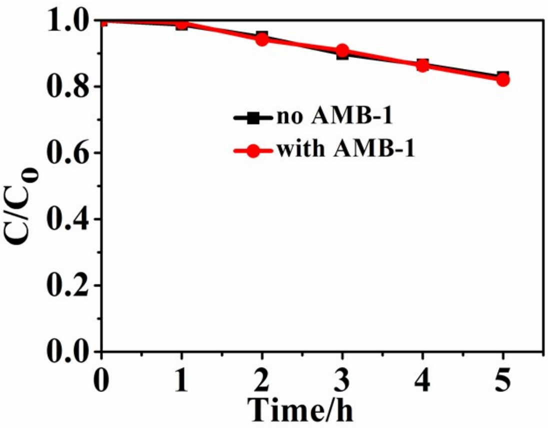

Fig. S3 Variation in concentration versus reaction time curves for H_2O_2 solution with/without AMB-1 nanoscale magnetic stirring bars under 45 °C.

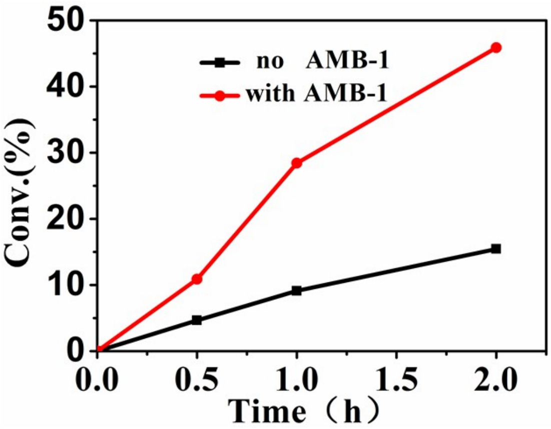

Fig. S4 Conversion versus reaction time curves for cyclooctene epoxidation with/without AMB-1 nanoscale magnetic stirring bars.
